# Quantitative analysis of residual protein contamination of podiatry instruments reprocessed through local and central decontamination units

**DOI:** 10.1186/1757-1146-4-2

**Published:** 2011-01-10

**Authors:** Gordon WG Smith, Frank Goldie, Steven Long, David F Lappin, Gordon Ramage, Andrew J Smith

**Affiliations:** 1 Institute of Infection, Immunity and Inflammation, Glasgow Dental School, College of Medicine, Veterinary and Life Sciences University of Glasgow, Glasgow, G2 3JZ, UK; 2 Central Decontamination Unit Cowlairs Industrial Estate 24 Finlas Street, Glasgow, G22 5DT, UK; 3 Podiatry Lead (North Acute) Department of Podiatry, Glasgow Royal Infirmary, Alexandra Parade, Glasgow G31 2ER, UK

## Abstract

**Background:**

The cleaning stage of the instrument decontamination process has come under increased scrutiny due to the increasing complexity of surgical instruments and the adverse affects of residual protein contamination on surgical instruments. Instruments used in the podiatry field have a complex surface topography and are exposed to a wide range of biological contamination. Currently, podiatry instruments are reprocessed locally within surgeries while national strategies are favouring a move toward reprocessing in central facilities. The aim of this study was to determine the efficacy of local and central reprocessing on podiatry instruments by measuring residual protein contamination of instruments reprocessed by both methods.

**Methods:**

The residual protein of 189 instruments reprocessed centrally and 189 instruments reprocessed locally was determined using a fluorescent assay based on the reaction of proteins with o-phthaldialdehyde/sodium 2-mercaptoethanesulfonate.

**Results:**

Residual protein was detected on 72% (n = 136) of instruments reprocessed centrally and 90% (n = 170) of instruments reprocessed locally. Significantly less protein (p < 0.001) was recovered from instruments reprocessed centrally (median 20.62 μg, range 0 - 5705 μg) than local reprocessing (median 111.9 μg, range 0 - 6344 μg).

**Conclusions:**

Overall, the results show the superiority of central reprocessing for complex podiatry instruments when protein contamination is considered, though no significant difference was found in residual protein between local decontamination unit and central decontamination unit processes for Blacks files. Further research is needed to undertake qualitative identification of protein contamination to identify any cross contamination risks and a standard for acceptable residual protein contamination applicable to different instruments and specialities should be considered as a matter of urgency.

## Background

The decontamination processes for medical instruments are under constant review as new challenges to instrument reprocessing emerge due to the increasing complexity of instruments and the emergence of variant Creutzfeldt Jackob disease (vCJD) which demonstrates reduced susceptibility to the common microbial inactivation processes [1]. Investigations into the biological properties of prion protein have highlighted the importance of the cleaning phase to remove protein and debris [2,3]. Moreover, the presence of residual protein on surgical instruments has been shown to increase the dissolution of metal ions, therefore increasing the rate of corrosion of certain instrument stainless steel [4]. In addition, residual protein may promote the adhesion of bacteria through specific adhesion receptors, such as fibronectin binding protein found in *Staphylococcus aureus *[5]. Protein can also inhibit sterilization processes if not removed during instrument cleaning [6].

Currently, the majority of podiatry instrument reprocessing is undertaken in local decontamination units (LDU). However, national strategies have favoured a predilection towards the centralisation of sterile services and the reprocessing of instruments at a central decontamination unit (CDU) [7]. CDU's offer the advantages of validated modern equipment, specialist knowledge, and shifts the legal responsibility of instrument reprocessing from the practitioner. Reprocessing in the LDU offers advantages with a faster instrument turnaround time and lower instrument inventory.

It is therefore important to determine the efficiency of the CDU process compared to current LDU processes at removing protein contamination to partly justify the change in strategy.

The aim of this study was to compare the efficacy of LDU and CDU reprocessing of podiatry instruments by a quantitative assessment of residual protein following routine use of the instruments.

## Methods

Pear burs (n = 126), Blacks files (n = 126) and Diamond Deb files (n = 126) manufactured by Timesco instruments UK were collected for the study after single use and randomly allocated into two groups for reprocessing. The first group was subjected to routine cleaning and sterilization by LDU's (Table 1) and the second group were subjected to reprocessing by the CDU at Cowlairs SSD Glasgow (Table 2). New, unused instruments representative of each type were also acquired from the manufacturers to serve as negative controls.

**Table 1:** Details of Podiatry LDU decontamination processes

Cleaning process	
Equipment	Hygena Ultrawave ultrasonic bath

Detergent	Sonozyme-solution changed twice daily

Cleaning time/temperature	6 mins/35°C

Validated	Tests and documentation supplied by manufacturer (Ultrawave)

**Sterilization Process**	

Equipment	Little sister 3 Type N (Non vacuum)

Method	Steam sterilization

**Table 2:** Details of Cowlairs CDU decontamination processes

Cleaning Process	
Equipment	Getinge Automated Washer Disinfector

Detergent	Dr Weigert Neodisher Mediclean Fort

Cleaning time/temperature	Pre rinse - 4 min 38 sec/Start 31°C End 34.9°C
	Main wash - 7 mins 20 sec/Start 60.5°C, End 62.8°C
	Hot water rinse - 2 mins/Start 91.4°C, End 92.6°C
	Disinfection - 1 min 30 secs 37
	Drying - 22 min 22 secs/Start 82.3°C, End 87.2°C

Validated	Washer disinfector by trust engineer to protocols defined in SHTM2030

**Sterilization Process**	

Equipment	Getinge Type B (Vacuum sterilizer)

Method	Steam sterilization

Individual Blacks and Diamond Deb files were placed in a sterile plastic bag (Seward, UK), whilst each Pear bur was added to a sterile 25 ml Universal tube (Corning, UK). Residual protein was desorbed from each instrument by immersion in a standardised volume of 1% v/v sodium dodecyl sulphate (SDS) (Sigma UK), and for Pear burs only the working end was immersed. Each instrument was subjected to sonication at 35 kHz for 30 min in an ultrasonic bath (Thermofisher Fisherbrand^® ^11021 sonic bath (Fisher Scientific, Loughborough UK)[8]. The protein desorbed from each instrument was subsequently quantified using a modification of the o-phthaldialdehyde (OPA)/sodium-2-mercaptoethanesulfonate assay, has a lower limit of detection of 5 μg/ml (See additional file 1). Briefly, the reagent was prepared by dissolving phthaldialdehyde (Sigma, Dorset UK) in methanol (BDH Laboratory supplies, Leicester, UK) to a produce a 300 mM solution. This was then added at a concentration of 1:50 into 1.2 M sodium 2-mercaptoethanesulfonate prepared in sodium tetraborate (100 mM [pH 9.2]). A 20 μl desorbed sample was added to a black Costar™ flat bottomed 96 well plate (Sigma, Dorset UK M9936) in combination with 300 μl of OPA reagent, as previously described by Zhu and colleagues [9]. The samples were incubated for 3 min at ambient room temperature before being measured using an Omega FluoStar plate reader (BMG Labtech, Aylesbury UK) at excitation wavelength 355 nm and emission wavelength 460 nm.

Data was analysed using SPSS (SPSS. Inc., Chcago, IL, USA) and the distribution of the data determined using the Kolmogorov-Smirnov test. The resultant non-parametric data was then compared using the Mann Whitney U test to analyse the differences between instruments reprocessed using the LDU and CDU, and to compare and analyse differences between each of the different instrument groups. The significance was determined by a 2-tailed Monte Carlo estimation.

## Results

**Figure 1:**
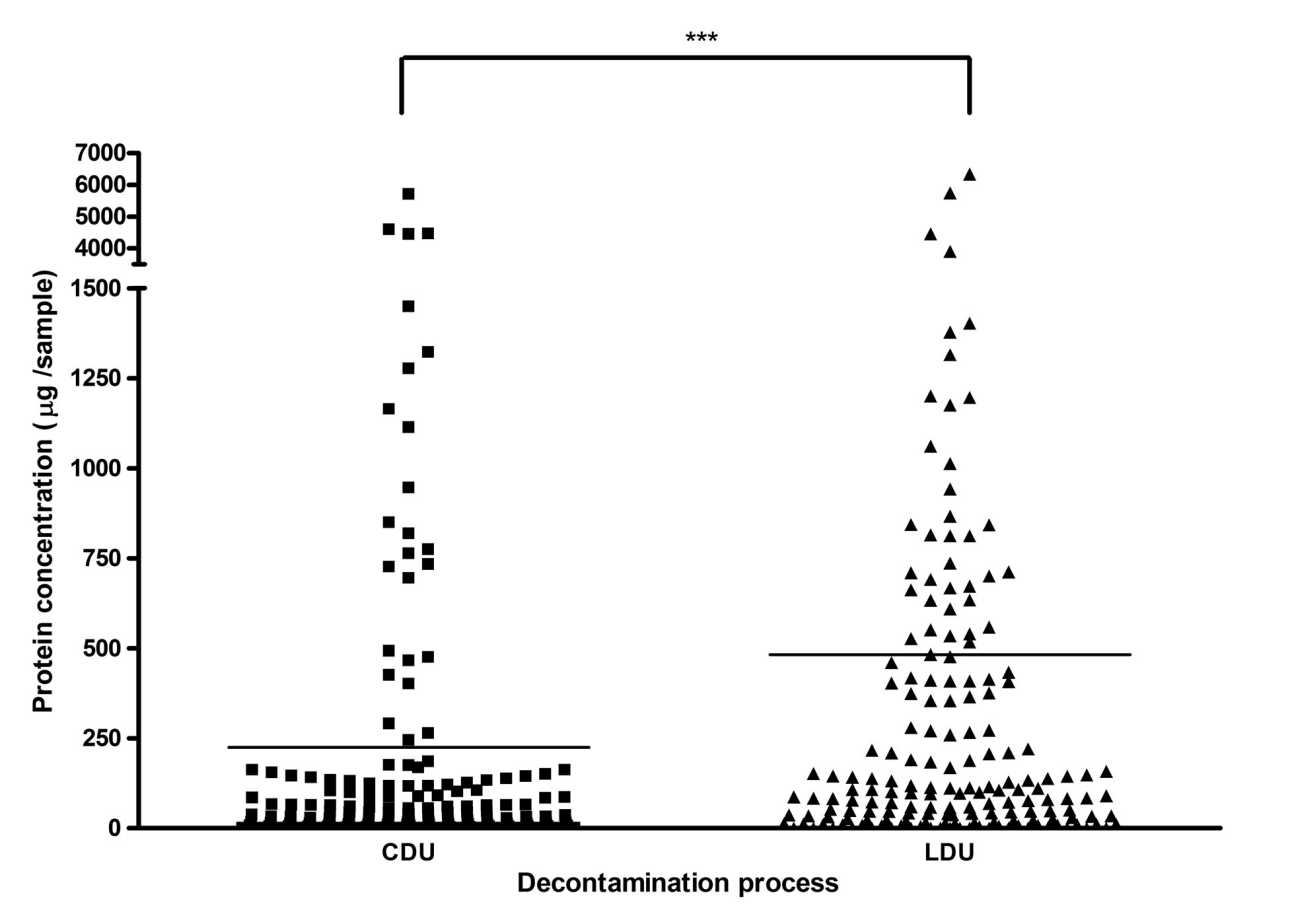
Residual protein isolated from all instruments after reprocessing by both methods (*** = P < 0.001).

A total of 58/63 Pear burs, 48/63 Blacks files and 31/63 Diamond Deb files reprocessed by CDU contained greater than 5 μg/instrument of detectable protein. Protein was also detected in 62/63 Pear burs, 53/63 Black files, and 56/63 Diamond Deb files reprocessed by LDU (Figure 1). Instruments reprocessed by the CDU (median 21 μg/instrument range 0-5705 μg/instrument) had significantly less residual protein than instruments reprocessed by the LDU (median117 μg/instrument range 0 - 6344 μg/instrument) when all three instruments were grouped (p < 0.001).

**Figure 2:**
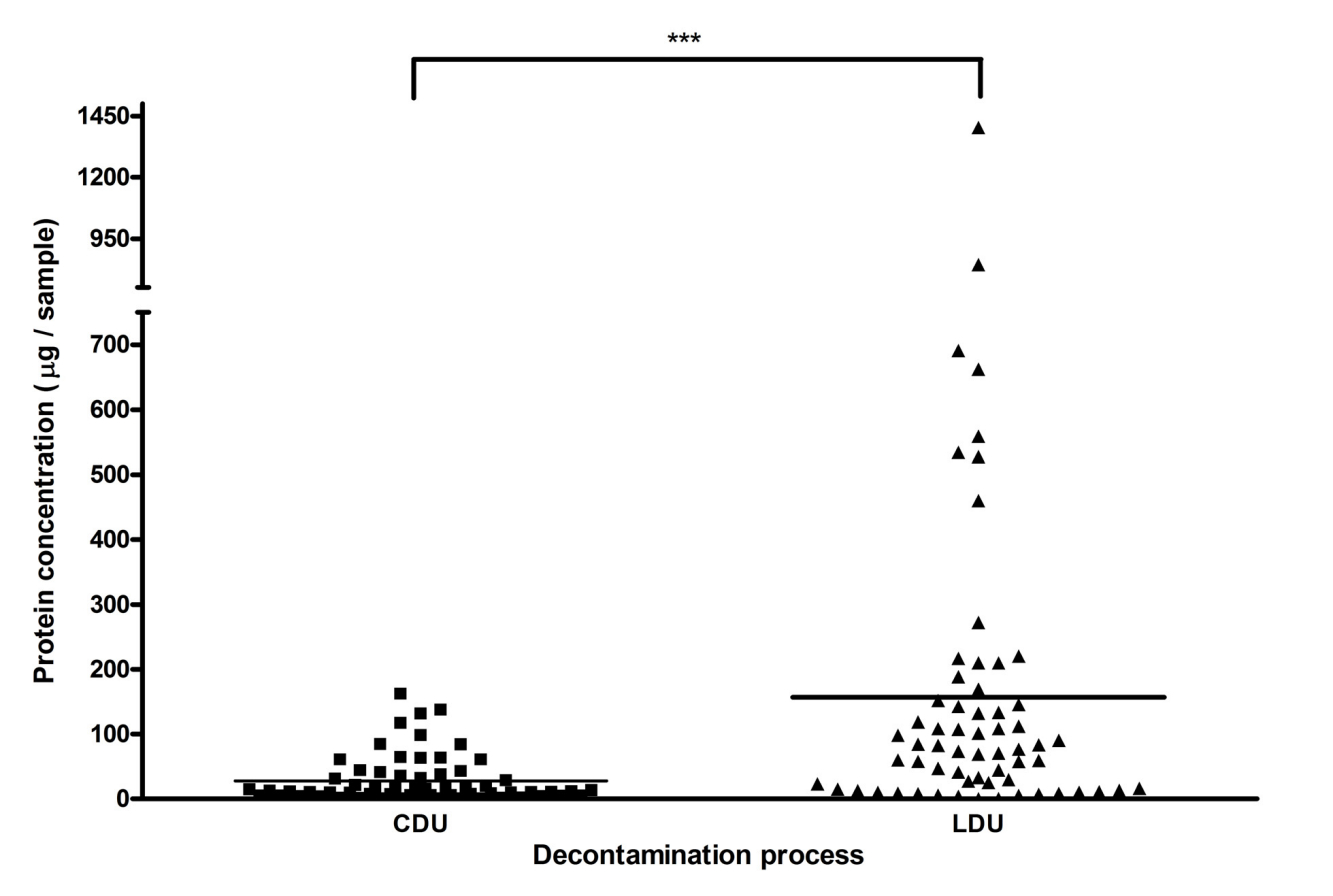
Total residual protein recovered from individual Pear burs reprocessed by both methods (*** = P < 0.001).

**Figure 3:**
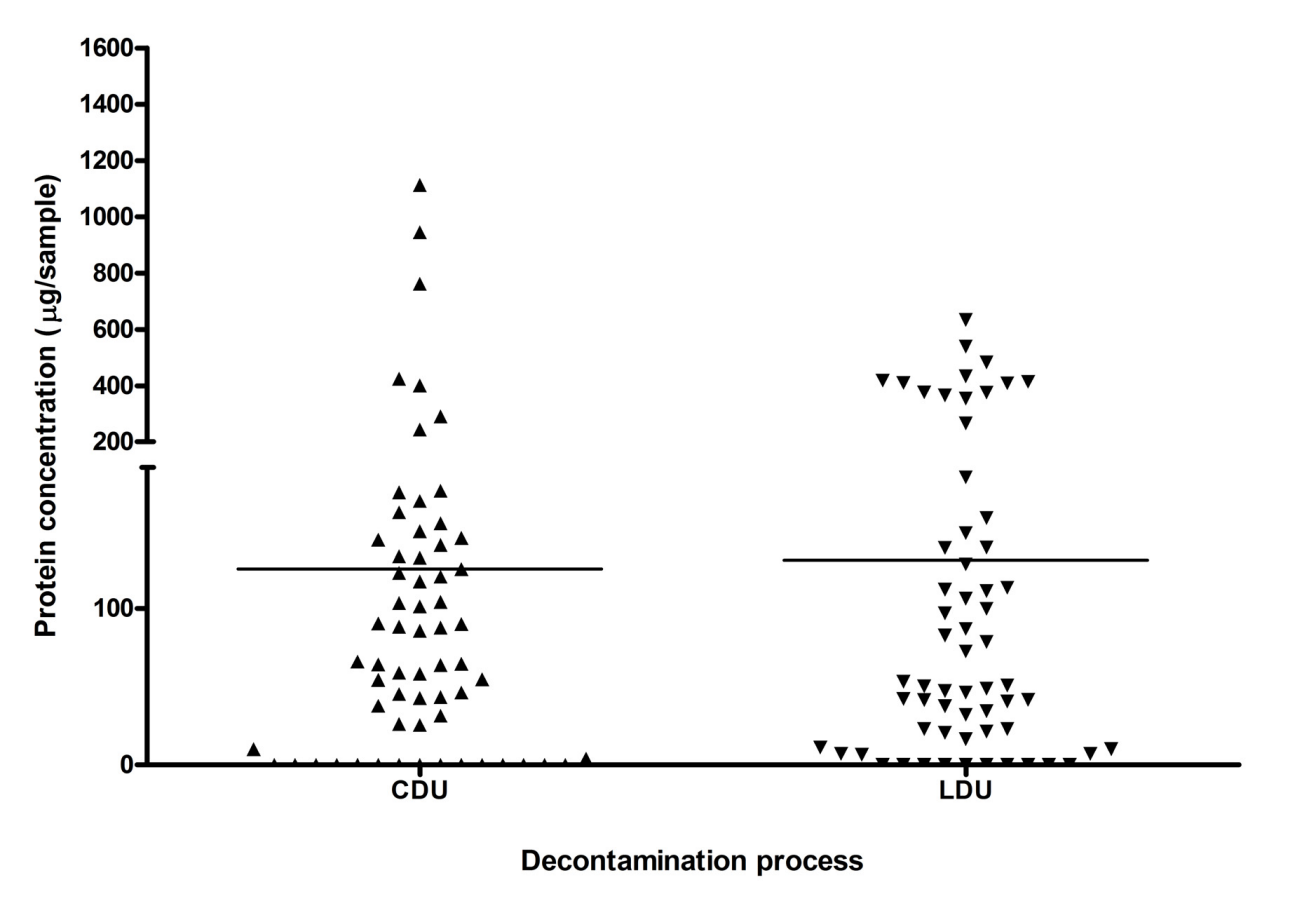
**Total residual protein recovered from individual Blacks files reprocessed by both methods**.

**Figure 4:**
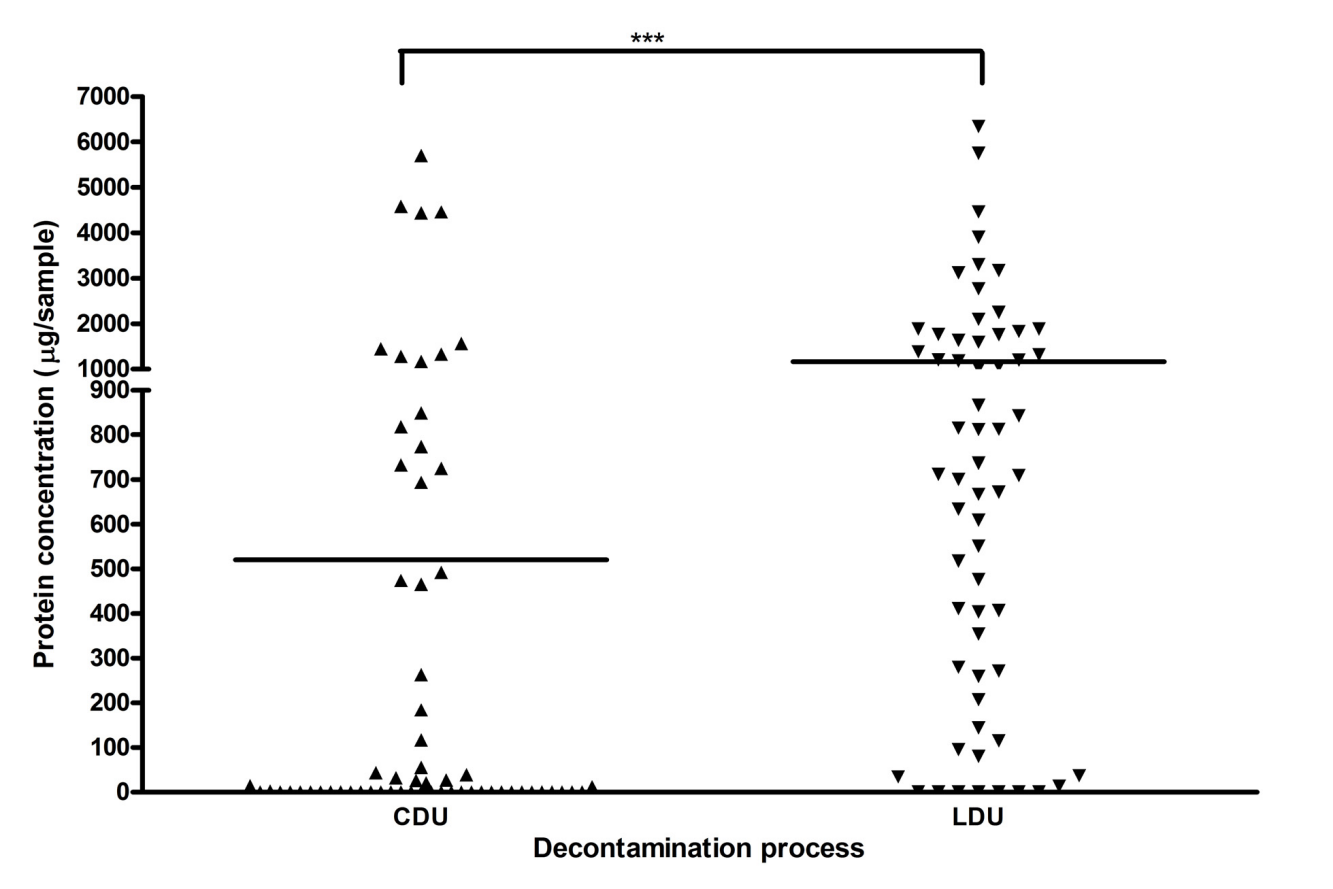
Total residual protein recovered from individual Diamond deb files reprocessed by both methods (*** = P < 0.001).

For individual instruments, the median quantity of protein detected on Pear burs (Figure 2) reprocessed by CDU was significantly lower (median 11 μg/instrument range 0-161.7 μg/instrument) than those by LDU (median 77 μg/instrument, range 0-1403 μg/instrument p < 0.001). The median quantity of protein detected on Blacks files (Figure 3) reprocessed by CDU (median 64.52 μg/instrument, range 0-1113 μg/instrument) exhibited no significant difference compared to protein detected on Blacks files by LDU (median 50.81 μg/instrument, range 0-633.5/instrument). The median quantity of protein detected on Diamond Deb files (Figure 4) reprocessed by CDU was significantly lower (0 μg range 0 - 5705 μg) than Diamond deb files reprocessed by LDU (median 711.8 μg, range 0 - 6344) (p < 0.05). However, residual protein was still detected from these instruments, as the mean of these was 512 μg for CDU reprocessing compared to 1159 μg for LDU reprocessing, indicating that a small proportion of CDU samples contained elevated levels of residual protein.

## Discussion

The cleaning stage of the medical instrument decontamination process has become increasingly important due to the emergence of (vCJD) and from the reported inhibition of the sterilization process caused by residual protein contamination [6]. Whilst there is an increasing trend for instruments to be reprocessed in centralised facilities, the majority of podiatry instruments are reprocessed locally. Concerns have been raised whether reprocessing in the LDU is less effective than the CDU for the decontamination of medical instrumentation [10].

This study was the first to directly compare the efficacy of CDU and LDU cleaning processes using podiatry instruments which were contaminated following routine use. When all podiatry instruments were grouped, the CDU instruments were found to contain significantly less residual protein than an identically sized group of instruments reprocessed by the LDU. The reason for the difference in cleaning efficacies between the CDU and the LDU are multifactorial and include a more robust validation process for the automated washer disinfectors (AWD) in use at the CDU. Other factors include an increased cleaning process time in the CDU (11 min- CDU compared to 6 min - LDU), different cleaning chemistries used, the differences in form of energy used in cleaning processes, and different temperatures used during the wash stage.

Similar patterns of cleaning efficacy were observed within each group of instruments with the exception of Blacks files, which may be due to the smaller ridged surface area compared to the more complex surface topography associated with the other instruments. This characteristic has been associated with increased retention of contamination by surface analysis of endodontic files which also have a ridged surface topography [11].

No single standard yet exists for "acceptable" protein levels on reprocessed instruments. The BS EN ISO-15883-1: 2006 for validation of washer disinfectors defines an acceptable level as below the detection limit of one of three protein assays which are stated as 2 mg/m^2 ^for the Ninhydrin assay, 30 - 50 μg for the bicinchoninic acid assay, and 0.003 μmol of OPA sensitive amino groups for the OPA assay [12]. Work undertaken by Lipscomb and colleagues (2006) also determined the threshold of sensitivity for similar reagents to be equivalent to 9.25 μg/10 mm^2^for Ninhydrin and 6.7 μg/10 mm^2 ^for the Biuret test [13]. Our group have determined a lower limit of detection for the OPA assay to be 5 μg/ml (see supplementary figure). If this was to be regarded as a threshold for cleanliness for reprocessed instruments, a total of 68/189 instruments reprocessed by CDU and 19/189 instruments reprocessed by the LDU would be deemed to be clean. The number of clean instruments may drop considerably if more sensitive analytical procedure were employed.

The data reported herein highlights the superiority of the CDU process in terms of cleaning efficacy at reprocessing more complex instruments. Previous studies have focused on the efficacy of CDU reprocessing by assaying a range of surgical instruments containing residual protein that was detected after reprocessing [8,14]. The protein content of different surgical instruments, including metzenbaum scissors and forceps, ranged from 163 to 756 μg, which is similar to that reported herein [14,15]. Similarly, a study on reprocessed dental endodontic files, which have a complex surface topography, showed a range of protein from 0.2 to 63.2 μg, similar to those levels observed on the Pear burs [8].

In order to improve validation of instrument reprocessing from visual inspection and published standards, techniques with greater quantitative sensitivity have emerged. Examples include a fluorescent microscopy technique involving visualisation of protein by SYPRO ruby staining capable of detecting 85 pg of protein on a surface area of 1 mm^2 ^which is significantly lower than the sensitivity of 5 μg/instrument reported in this study [10]. A standard for cleanliness when considering protein contamination should be dependent on the procedures undertaken by the instrument. The total protein recovered from the podiatry instruments would be equivalent to a large number of prion infectious units [13].

## Conclusions

Residual protein has been recovered from podiatry instruments reprocessed by the CDU and the LDU. This study has shown that overall, the CDU is superior to the LDU with respect to podiatry instrument reprocessing and that the level of complexity of the instrument may dictate the level of reprocessing for example the adoption of a single use policy or enhanced cleaning validation processes for certain instrument designs. Further studies are required to evaluate the reprocessing of a range of medical instruments using similar methodologies to those employed within this study, which will help validate these data. Moreover, understanding which proteins are associated with instruments is of critical importance, as this will have implications with regards to safety and risk assessment.

## Competing interests

The authors declare that they have no competing interests.

## Authors' contributions

GWGS carried out the processing and the subsequent protein analysis of all the instruments and for the overall study design and for the drafting of the manuscript. FG and SL were responsible funding of the chemicals used in the study, the sourcing of the instruments from community podiatry and the CDU and for helping in drafting the manuscript. DL carried out the statistical analysis and aided in study design. GR and AJS were responsible for the overall design of the study and aided in the final drafting of the manuscript. All authors have read and approved the final manuscript.

## Supplementary Material

Additional file 1 **Method validation**. Details of the methods and the results of validation experiments for the protein detection and protein extraction methods used in this study. Click here for file
