# Supplementary material for: Quantitative analysis of residual protein contamination of podiatry instruments reprocessed through local and central decontamination units
Source: J Foot Ankle Res. 2011 Jan 10;4:2. doi: 10.1186/1757-1146-4-2 (PMC3023740; doi:10.1186/1757-1146-4-2)
Supplement: Additional file 1 — Method validation. Details of the methods and the results of validation experiments for the protein detection and protein extraction methods used in this study. [file 1757-1146-4-2-S1.DOCX]

### Title: Supplemental details for materials and methods (ms:1266526906468429)

### Ophthaldialdehyde assay

The ophthaldialdehyde (OPA) reagent was prepared by adding 40 mg of phthaldialdehyde dissolved in 1 ml of methanol (BDH Laboratory supplies, Leicester, UK) to a solution of 100 mg of mercaptoethanesulfonate dissolved in 50 ml of 0.1M pH 9.2 sodium tetraborate. A 20µl protein sample was added to a Costar™ dark flat bottomed 96 well plate (Sigma, Dorset UK) and 300 µl of OPA reagent was added to each sample. The samples were incubated for 3 min at ambient room temperature before being sampled using an Omega Fluostar plate reader (BMG Labtech, Aylesbury UK) at excitation wavelength 355 nm and emission wavelength 460 nm[9].

###

### Protein standard curves

A standard curve of protein concentrations 1, 5,10,20,50,100 µg /ml of protein was prepared in RO H_2_O. A blank control of RO H_2_O was also included in standard curves when used to determine the sensitivity of the protein assays and values of unknown samples.

### Validation of protein extraction methods

Medical grade stainless steel discs that had been prepared as described previously (Section) were inoculated with a 10 µl solution of 10 mg/ ml BSA for a final concentration 100 µg / ml solution on the disc. Discs were then dried for 16h overnight at ambient room temperature. Discs were subjected to one of the following treatments; sonication for 30 mins in Decon 90 (Decon laboratories East Sussex UK) detergent, sonication for 30 mins in 1% SDS, and boiling for 10 mins in 0.1M NaOH. Each disc was inserted into a sterile 10 ml Bijoux (place name) and immersed in 1 ml of the appropriate detergent; turbines were inserted into a 25 ml Universal tube and immersed in 2 ml of the appropriate detergent. For sonication, each tube was inserted into a Fisherbrand^®^ 11021 sonic bath (Fisher Scientific Leicestershire UK) and subjected to sonication at 35 kHz for the appropriate time. For boiling, the tubes were inserted into a water bath (Grant Instruments) set at 100 °C for the appropriate time. A sample of the eluent was taken for the OPA assay and sampled as previously described . Discs were inoculated with RO H_2_O and dried for 16h as negative controls. A sample was also taken for SDS – polyacrylamide gel electrophoresis (PAGE).

### Results

### Protein assay standard curves

An increase in BSA protein concentration results in a corresponding linear increase in fluorescent units (figure 1) for the OPA assay with the limit of detection being 5 µg /ml.

Figure 1 OPA Bovine serum albumin standard curves diluted in1 % SDS. The BSA standard. The data shown is the average of 3 readings from 3 experiments ± the SEM. The r^2^ value is calculated as 0.9834.

### Validation of protein extraction methods

The method that recovered the most protein was sonication in 1% SDS (Table 1). Boiling in 0.1M NaOH recovered the least protein. BSA extraction by sonication in 1% (v/v) was visualised by SDS-PAGE (Figure 2).

Table 1 Efficacy of protein extraction techniques and detergents. The data shown is the median of three experiments.

| Detergent | Extraction Process | Median protein recovery (µg / ml) | Range of protein recovery (µg / ml) |
| --- | --- | --- | --- |
| 1 % (v/v) SDS | Sonication | 80 | 63.7 – 94.9 |
| 1 % (v/v) Decon90 | Sonication | 57 | 22.8 – 77.8 |
| 0.1M NaOH | Boiling | 53 | 50 – 66.5 |


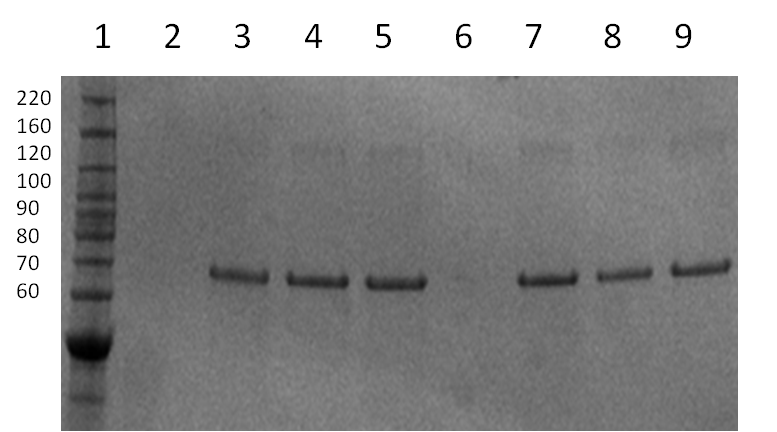


Figure 2 Recovery of BSA by sonication and boiling technique. Both boiling (lanes 3 ,4 ,5) and sonication (lanes 7, 8, 9) recover BSA from stainless steel discs. No protein is visible in the negative controls (lane 2 and lane 6).
